# Morphological and optical data of AgNW embedded transparent conductive layer

**DOI:** 10.1016/j.dib.2016.08.060

**Published:** 2016-09-04

**Authors:** Hong-Sik Kim, Dipal B. Patel, Malkeshkumar Patel, Joondong Kim

**Affiliations:** Photoelectric and Energy Device Applications Lab (PEDAL) and Department of Electrical Engineering, Incheon National University, 119 Academy Rd. Yeonsu, Incheon 406772, Korea

**Keywords:** AgNWs, ITO thin films, Thermal processing, Optical properties

## Abstract

In this data article, morphological and optical data of AgNW encapsulated between ITO layers are presented to get insights into our article (DOI:10.1016/j.solmat.2016.04.038; Hong-Sik Kim, Pankaj Yadav, Malkeshkumar Patel, Hyunki Kim, Kavita Pandey, Joondong Kim, 2016) [1]. SEM images for the formation of AgNWs networks by number of spin coating are also presented. SEM photographs showing the surface morphologies before and after rapid thermal treatment of prepared samples have been presented. Apart from morphological data set, optical characteristics of this type of samples are given. The comparison plots of optical reflectance from AgNW encapsulated between ITO layers and bare ITO are given between the wavelength ranges from 300 to 1100 nm. At the end, transmittance and reflectance curves of native glass substrates used in this study are presented.

**Specifications Table**

**Table:** 

Subject area	*Physics*
More specific subject area	*Solar cells*
Type of data	*Figures*
How data was acquired	*SEM (JSM-70001F, JEOL) and UV-visible spectrophotometer (UV-2600, Shimadzu)*
Data format	*Analyzed*
Experimental factors	*Prepared AgNW coated ITO samples were treated under rapid thermal processing (RTP) to observe the morphologies before and after annealing*
Experimental features	*Progressive growth of AgNW by multi-coating approach*
Data source location	*Incheon National University, Incheon-406772, Korea*
Data accessibility	*The data are with this article*

**Value of the data**

• Analyzed surface morphologies of AgNW and AgNW encapsulated between ITO layers before and after Rapid Thermal Processing (RTP) would be useful for tunning the required morphological characteristic of various TCO materials.
• The number of spin coatings can be treated as a function to modulate the packing density of nanostructures (like AgNW) on the substrates.
• Remarkable improvement in the reflectance property of AgNW encapsulated ITO layer between the wavelength ranges from 320 to 510 nm suggest a route to selectively improve the optical characteristics of particular TCO material.
• The data will be useful in understanding the role of AgNW in transparent conducting contacts and designing of solar cell.

## 1 Data

**Fig. 1:**
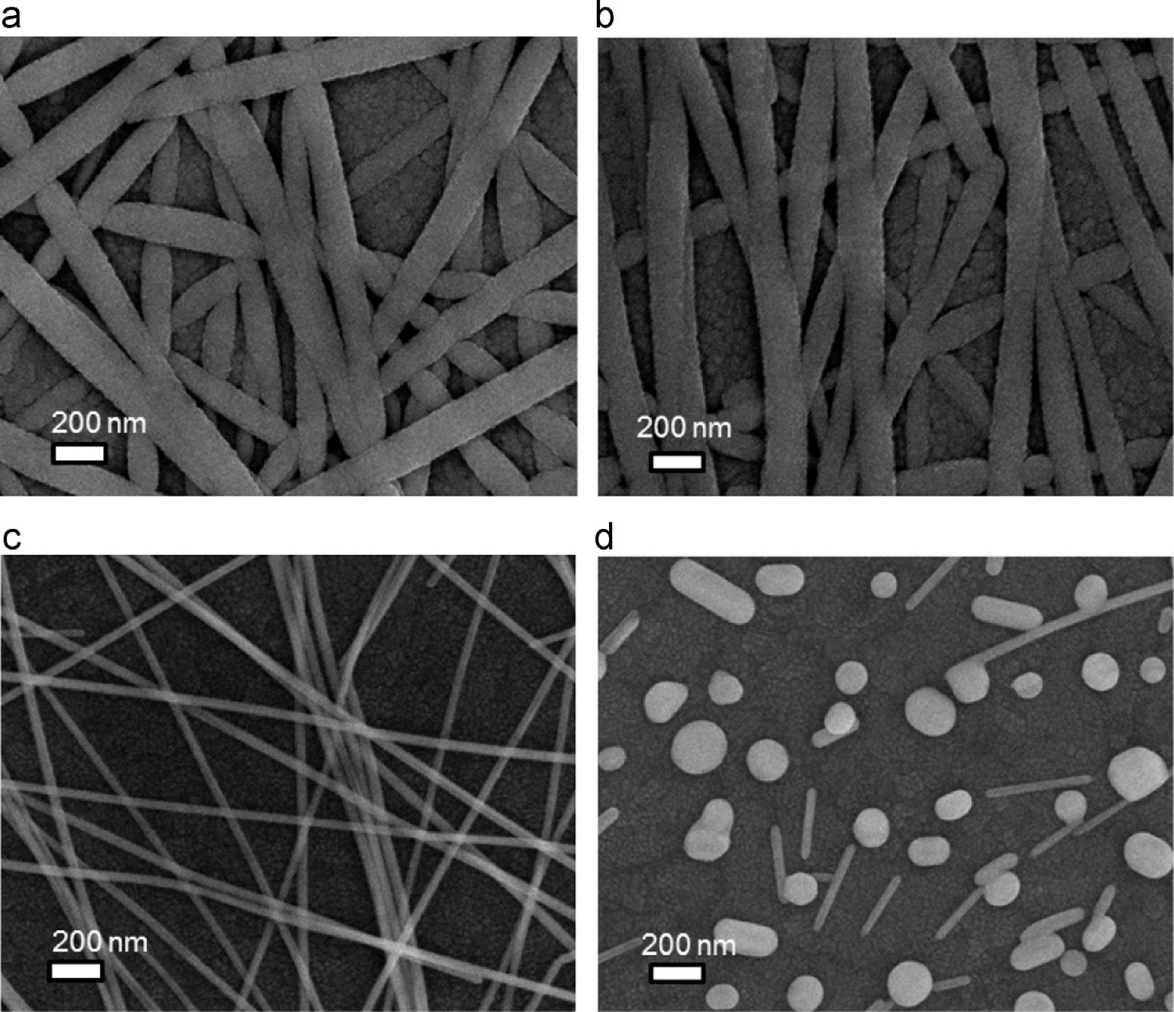
SEM images of ITO/AgNWs/ITO (a and b) and AgNW (c and d), before (a and c) and after (b and d) RTP process, respectively.

**Fig. 2:**
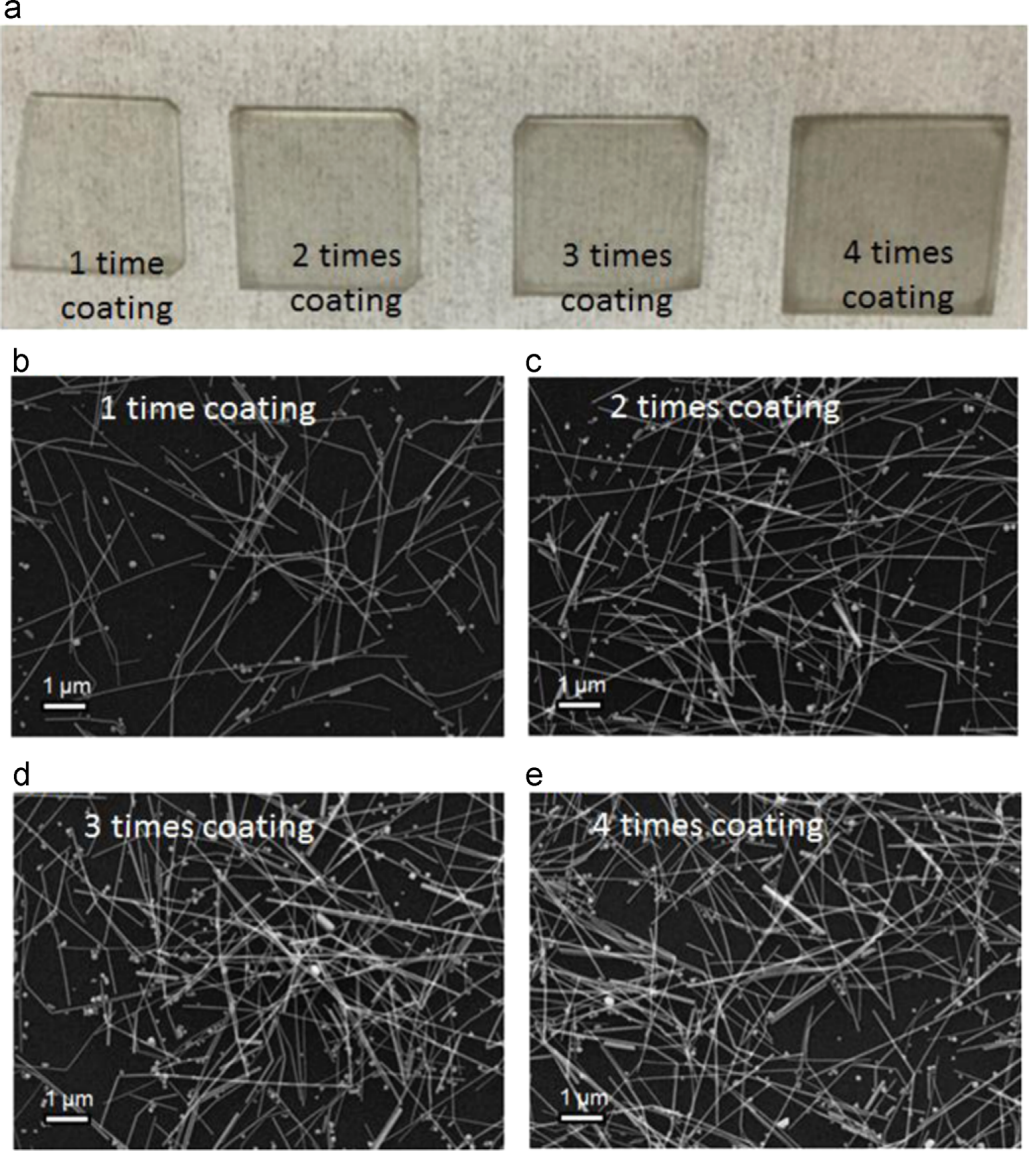
(a) Photographic image and (b) SEM images of AgNW coated ITO samples after each coating.

**Fig. 3:**
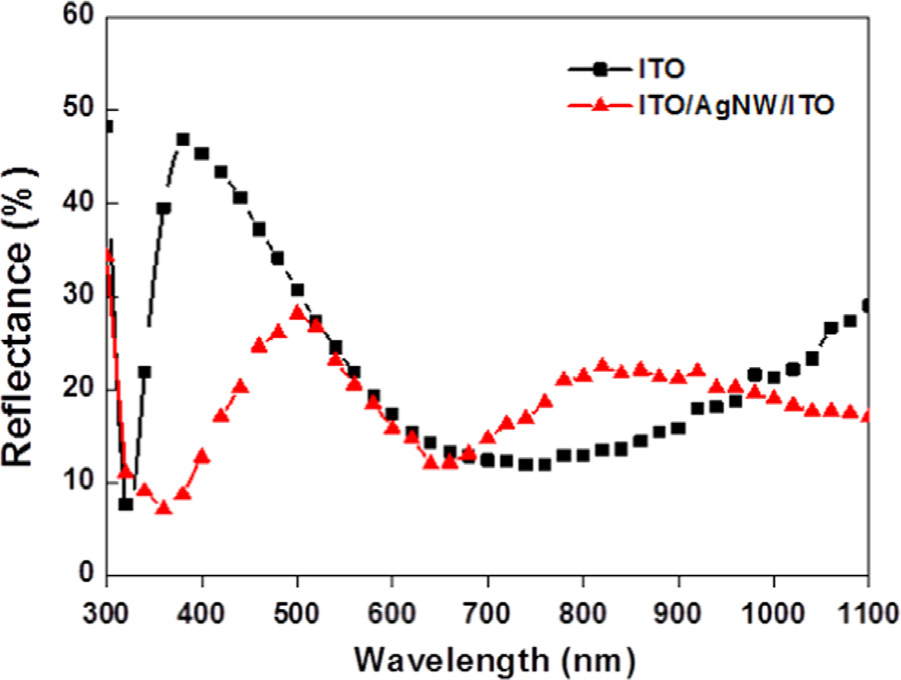
Reflectance of ITO and AgNWs embedded ITO on Si wafer.

**Fig. 4:**
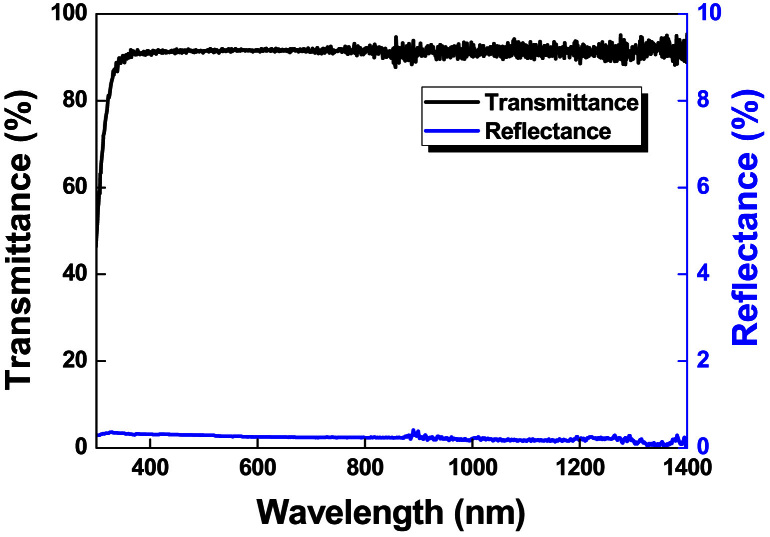
The Transmittance and reflectance spectra of native glass substrates.

The morphological and optical data sets are acquired from the fabricated samples. Fig. 1 shows surface morphologies recorded by using scanning electron microscope (SEM) of the samples before and after RTP treatment. Photographic and SEM images of prepared samples by multiple coating of AgNW on soda lime glass substrates are presented in Fig. 2(a) and (b), respectively. Reflectance and transmittance data of fabricated samples and glass substrate are given in Fig. 3, Fig. 4, respectively.

## 2 Experimental design, materials and methods

### 2.1 Sample preparation

The sample preparation included the experiments for deposition of AgNW on and in between ITO layers followed by post annealing through RTP technique. A uniform 100 nm thick ITO layer was first deposited on Si and soda lime glass substrates using DC sputtering [1]. Upon depositing underneath ITO layer, a spin coating was utilized to spread-out the ink solution of AgNW assemblies all over the ITO layer [1]. On the top of the AgNW matrix, another 100 nm thick layered ITO deposition was done under same sputtering conditions. A parallel batch of samples, containing uniform thin film of ITO of 200 nm thickness was also prepared for comparison purpose. The prepared batches of samples are further annealed in controlled RTP unit at 500 °C. In the second set of experiments, the number of AgNW spin coatings was varied from 1 to 4 spins on soda lime glass and ITO substrates [1].

### 2.2 Sample characterizations

Surface morphologies of the newly architecture TCO (AgNW embedded ITO) and dispersed AgNWs on ITO surface are shown in Fig. 1. These samples were thermally treated at 500 °C to observe morphological changes of AgNWs. Fig. 1 shows SEM images before (a and c) and after (b and d) thermal treatments. A photographic and SEM images taken after each run of spin coating AgNWs assembly on glass/ITO substrates are presented in Fig. 2(a) and (b), respectively. The packing density of dispersed AgNWs was controlled by number of spin-coating.

Reflectance data for the fabricated samples between the wavelength range of 1100–300 nm are presented in Fig. 3. An integrated sphere attachment supplied with UV–vis spectrophotometer (Shimadzu-2600) was used for carrying out the diffused reflectance measurements. A necessary baseline correction was done prior to recording the reflectance spectra by using BaSO_4_ pallets. The transmittance and reflectance characteristics of bare glass substrate are given in Fig. 4. While recording the transmittance spectrum of glass substrate, a baseline correction with reference to the air was utilized.
