# Supplementary material for: Morphological and optical data of AgNW embedded transparent conductive layer
Source: Data Brief. 2016 Sep 4;9:177–82. doi: 10.1016/j.dib.2016.08.060 (PMC5021792; doi:10.1016/j.dib.2016.08.060)
Supplement: Supplementary file 1 — Supplementary material [file mmc1.docx]

***Conflicts of Interest Statement***

Re: DIB-D-16-00314 (MS-SOLMAT-D-15-01571, PII-S0927-0248(16)30061-7)

Title: *Morphological and optical data of AgNW embedded transparent conductive layer*

We declare that this manuscript is original, has not been reported before, and is not currently being considered elsewhere. We also confirm that there is no known conflict of interest regarding this manuscript and its publication. The manuscript has been approved by all named authors.

Sincerely yours,


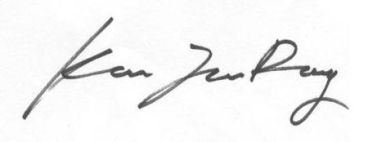


Joondong Kim

Joondong Kim, Ph.D./Professor

Department of Electrical Engineering,

Incheon National University

E-mail: joonkim@ incheon.ac.kr

Phone: +82-32-835-8770; fax: +82-32-835-0773
